# Spanish Validation of the Multidimensional Existential Meaning Scale: Which Dimension of Meaning in Life Is More Associated With Psychopathology in People With Mental Disorders?

**DOI:** 10.3389/fpsyt.2022.832934

**Published:** 2022-02-02

**Authors:** Jose Heliodoro Marco, Joaquín García-Alandete, Sandra Pérez Rodríguez, Verónica Guillén, Rosa M. Baños, Maria Pilar Tormo-Irun

**Affiliations:** ^1^University of Valencia, Valencia, Spain; ^2^CIBER Fisiopatología Obesidad y Nutrición (CIBEROBN), Madrid, Spain; ^3^Valencian International University, Valencia, Spain

**Keywords:** meaning in life, coherence, purpose, mattering, positive affect

## Abstract

**Background:**

To assess three dimensions of Meaning in Life (comprehension, purpose, and mattering) the Multidimensional Existential Meaning Scale (MEMS) was developed, however, the MEMS's factorial structure has not yet been confirmed in a Spanish-speaking sample. A question that remains unanswered is which of the three dimensions of MiL are associated with psychopathology in clinical samples.

**Aims:**

(1) to analyze the psychometric properties of the MEMS in a Spanish non-clinical population, and (2) to identify which of the three dimensions of MiL shows the strongest relationship with depression, anxiety and positive affect in a clinical population.

**Method:**

The non-clinical sample, consisted of *N* = 1106 Spanish adults, and the clinical sample consisted of 88 adults diagnosed with mental disorders. A Confirmatory Factor Analysis and regression analysis were carried out.

**Results:**

The three-factor model for the MEMS showed an acceptable fit, and full invariance across gender groups. In the clinical sample, the mattering dimension had the highest association with depression and anxiety, and purpose with positive affect.

**Conclusion:**

The MEMS is an adequate instrument to assess the three dimensions of meaning in Spanish-speaking participants. These results support the importance of evaluating the MiL construct from a multidimensional perspective in clinical samples.

## Introduction

Meaning in life (MIL) can be defined as the extent to which one's life is experienced as making sense, being directed and motivated by important goals, and mattering in the world. Several scales have been developed to assess MiL, both unidimensional scales, such as the Purpose in Life scale (PIL) (1), and multidimensional scales, such as the Meaning in Life Questionnaire (MLQ) (2). Multidimensional models seem to assess MiL more accurately than unidimensional models by distinguishing different facets or dimensions of MiL. Even the PIL, a classic instrument for the assessment of MiL, has shown better psychometric properties and been found to be more clinically useful when different dimensions have been distinguished (3). Therefore, it appears that multidimensional models for the assessment of MiL are preferrable to unidimensional models.

Martela and Steger (4) elaborated a tripartite conceptualization of MiL, suggesting that MiL would be made up of three clearly interconnected dimensions that interact with each other, making us feel that life has meaning: Comprehension, purpose, and mattering. The comprehension dimension refers to the extent to which people feel their life is coherent, predictable, and connected as a whole (5, 6). Thus, comprehension is the cognitive dimension of MiL. Low comprehension of one's life means that the person feels that his/her life is incoherent, fragmented, and confused. An example would be the feeling of low comprehension that occurs after experiencing a traumatic event that has violated one's schemes about the world and one's life. The violation of these schemes would reduce comprehension and increase distress (7). In contrast, people with high comprehension feels that their life is meaningful, that everything happens in a coherent way, and that the positive or negative events that occur daily are integrated into their global schemes. Previous studies found that the meaning-making process moderates and mediates several mental disorders, such as posttraumatic stress disorder (8), eating disorders (9), adjustment disorders (10), and stress (11).

The purpose dimension refers to the extent to which a person's life is aimed at achieving specific goals and values (12), and the extent to which the person takes responsibility for these goals. Thus, purpose is the motivational dimension. This dimension was originally proposed by Viktor Frankl (13), who suggested that human beings should be directed toward something or someone greater than themselves that transcends them, in order to experience MiL. High-purpose people feel that their life is oriented toward functional, adaptive goals that extend beyond themselves. These people usually develop creative goals (doing something, work, academic career, hobbies, among others) and experiential goals (loving something or someone, feeling, caring, interpersonal relationships, among others). Low-purpose people feel that their life is not oriented toward functional or important goals for them, or they feel that their life has no purpose. The dimension of purpose has been negatively associated with borderline personality disorder psychopathology (14), hopelessness (15), and mortality risk (16).

The mattering dimension, also known as significance (4), refers to the extent to which people feel that their existence is important and significant and has intrinsic value to the world (17). People high in mattering think that they are valuable for merely existing, that life has intrinsic value, and that if they did not exist the world would have been different. People feel that their life matters because it is important to the people around them (e.g., interpersonal relationships). When people experience mattering, they feel that their actions and existence make a difference in the world around them and that their lives are valuable (18). Thus, mattering is based on a global evaluation of one's life from a spiritual or existential perspective. Mattering is the affective dimension of MiL, and it has been found to be positively associated with positive affect, self-esteem, and well-being (19), and negatively associated with hostility, aggression, and negative affect (20, 21).

Although mattering is the dimension of MiL that has been investigated least and, to our knowledge, there are no studies with clinical populations, there has recently been an increased interest in this dimension and its role in the MiL construct. Costin and Vignoles (18), in a longitudinal study with a non-clinical population, analyzed which of the three dimensions of meaning (coherence, purpose, and mattering) made a greater contribution to the feeling of MiL, and they found that mattering was the greatest predictor of MiL. These results suggest the need to use instruments that measure the three dimensions of meaning and analyze their differential contributions to psychopathology and mental health.

To assess the comprehension, purpose, and mattering dimensions of MiL George and Park (22) developed the Multidimensional Existential Meaning Scale (MEMS). To develop the MEMS, 43 items were generated to capture the three dimensions of MiL. These items were qualitatively examined by several experts in measuring MiL, and 29 items were retained. Three samples of university students from the United States were utilized to perform several exploratory and confirmatory factor analyses (CFA), and the MEMS was reduced to 15 items to evaluate the three dimensions of MiL: items 1, 7, 8, 10, and 14 assess comprehension; items 3, 5, 6, 9, and 12 assess purpose; and items two (which is reverse-scored), 4, 11, 13, and 15 assess mattering. The MEMS subscales showed good internal consistency, and good validity (the subscales showed strong associations with the Presence subscale of the MLQ). Moreover, the MEMS subscales were associated with well-being variables, such as positive affect. Comprehension had the highest negative association with psychopathology constructs (depression, anxiety, and negative affect), and purpose had the highest positive association with positive affect.

In a sample with 401 Polish participants a CFA was performed on the Polish version of the MEMS confirmed the reliability and validity of the trifactorial structure of a reduced scale containing nine items (23). The Comprehension factor was composed of items 7, 8, and 10; the Purpose factor consisted of items 3, 5, and 9; and the Mattering factor contained items 2, 13, and 15. Finally, the three subscales were highly and positively associated with MiL, assessed with the PIL and the MLQ Presence subscale.

In summary, the MEMS has been shown to be a reliable and valid instrument to assess the three dimensions of MiL (comprehension, purpose, and mattering) in English- and Polish-speaking samples. However, its factorial structure has not yet been analyzed and confirmed in a sample of Spanish-speaking people. Moreover, previous studies have associated the three dimensions of MiL with psychopathology variables however they have always been carried out in non-clinical populations (2). In addition, a question that remains unanswered is which of the three dimensions presents a greater association with psychopathology and positive affect in patients diagnosed with mental disorders. The identification of these relationships would allow a better understanding of the functioning of these variables as protective factors that can help to improve prevention or treatment programs for some mental disorders.

The present study has two aims: (1) to analyze the psychometric characteristics and confirm the factorial structure of the MEMS in a Spanish non-clinical population (Sample 1); and (2) to identify which of the three dimensions of MiL, assessed with the MEMS (comprehension, purpose, or mattering), shows the strongest relationship with psychopathological distress and positive affect in a clinical population (Sample 2).

## Materials and Methods

### Participants

The non-clinical sample (Sample 1) consisted of *N* = 1106 Spanish participants between 18 and 83 years old (*M* = 35.05; *SD* = 13.72); 80.4% were women. The majority of the participants were married (51.1%) or single (42%), 5.9% were separated or divorced, and 1% widowed. Regarding their level of education, 49.6% of the sample had a university degree, 33.1% had a university master's degree, 16.3% had secondary studies, and 1% had primary studies. All participants were Spanish. The exclusion criterion was having been diagnosed with any mental disorder.

The clinical sample (Sample 2) consisted of 88 participants with a mean age of 29.34 (*SD* = 11.95), and a range of 19–67 years; *n* = 80 (90.9%) were women. The inclusion criteria were being an adult, having a diagnosis of a mental disorder, and receiving psychiatric or psychological treatment at the time of the evaluation. Regarding the residence country, 52 participants (59.1%) were from Spain, and 33 participants (40.9%) were from other Spanish-speaking South American countries. Regarding marital status, 54 participants (60.4%) were single or separated, and 34 participants (38.6%) were married. Most of the participants (*n* = 68, 77.3%) did not have children, and 38.6% were employed.

Regarding clinical characteristics of the sample, 88 participants (100%) were undergoing pharmacological or psychological treatment, 42 participants (47.72%) were diagnosed with anxiety disorders, 24 participants (27.27%) were diagnosed with depressive disorders, nine participants (10.22%) were diagnosed with bipolar disorders, nine participants (10.22%) were diagnosed with obsessive compulsive disorder, and four participants (4.54%) were diagnosed with posttraumatic stress disorder.

### Instruments

*The Multidimensional Existential Meaning Scale* (MEMS) (22) assesses the MiL dimensions: Comprehension, purpose, and mattering, with a total of 15 items (e.g. “My life makes sense”; “I have overarching goals that guide me in my life”; “I understand my life”; “I know what my life is”). Likert type responses are given on a seven-point scale (1 = Very strongly disagree; 7 = Very strongly agree). This self-report is described in the introduction section. In our sample, the three MEMS subscales showed adequate internal consistency: Comprehension ($\bar{\omega }$ = 91), Purpose ($\bar{\omega }$ = 92), and Mattering ($\bar{\omega }$ = 86). For this study, the MEMS was translated from English to Spanish by two PhD researchers who are experts in MIL assessment, and subsequently a back translation from Spanish to English was carried out by two other expert PhD researchers. The whole translation and back translation process were overseen by a bilingual native English editor.

*The Brief Symptom Inventory 18* [BSI-18; (24, 25)]. The BSI-18 was designed to assess psychopathological distress. This instrument consists of 18 items rated on a five-point Likert scale (0 = Not at all; 4 = Extremely) indicating the presence of the symptom in the past seven days. The BSI-18 yields a global score, the General Severity Index, and three subscale scores: somatization, depression, and anxiety. In this study, we used the depression, anxiety subscale and the General Severity Index. In the present study, excellent internal consistency was found for the BSI-18 total scale ($\bar{\omega }$ = 0.96).

*The Positive and Negative Affect Schedule* [PANAS; (26, 27)]. The questionnaire includes 20 adjective items, 10 assessing positive affect (PANAS-P) and 10 assessing negative affect (PANAS-N). Respondents are asked to rate the extent to which they had experienced each particular emotion within a specified time period, using a five-point scale (1 = Very slightly or not at all; 5 = Very much). In this study, both affect subscales showed adequate internal consistency: PANAS-P, $\bar{\omega }$ = 0.92, and PANAS-N, $\bar{\omega }$ = 0.88.

*Purpose In Life Test-10* [PIL-10; (28)]. This scale is a reduced Spanish version of the PIL (29), and it is composed of a 10-item Likert scale related to different aspects of meaning in life (e.g., “In life I have many definite goals and longings”). The PIL-10 has two subscales. The total score ranges from 10 to 70: higher scores indicate greater MiL. In this study, the PIL-10 showed adequate internal consistency ($\bar{\omega }$ = 0.94).

### Procedure

For the present study, two samples were selected: Sample 1 was composed of non-clinical participants, and Sample 2 was composed of clinical participants. For the two samples, we used snowball sampling techniques to recruit participants through main social networks (Facebook, WhatsApp, Twitter, Linkedin, and Instagram) and a massive mailing to the researchers' contacts in May and July 2020. In Internet announcements, we described the study and requirements for participation. All participants provided their consent to participate in the study, and they answered a 20-min survey using the Google Forms online platform. The inclusion criteria were being 18 years old, speaking Spanish, and signing the informed consent. In this online survey, the following question appeared: “Are you currently diagnosed with a mental disorder?” “Indicate which one”; and “Are you in psychiatric or psychological treatment for your mental disorder at this time?” yes/no. If the participants did not have any diagnosis and were not receiving psychiatric or psychological treatment, we considered them for the non-clinical sample. If, on the other hand, the participants had a diagnosis of a mental disorder and were currently undergoing psychiatric or psychological treatment, they were part of the clinical sample. Participants did not receive any compensation for participating in the study.

### Data Analyses

With the participants in the non-clinical sample, we carried out the following analyses: First, descriptive statistics, skewness, kurtosis, and internal consistency (McDonald's ω) of the scales used in this study were calculated. Second, descriptive statistics of the MEMS items as well as the item-rest correlations and the average inter-item correlations of the MEMS subscales were calculated. Third, a Confirmatory Factor Analysis (CFA) was carried out to evaluate the structural validity of the MEMS, and a Multi-Group Confirmatory Factor Analysis (MG-CFA) was performed to evaluate the structural invariance of the MEMS subscales across gender and age groups (30). Because Mardia's coefficient, normalized estimate, was higher than five (that is, multivariate normality was not assumed) and the MEMS subscales are ordinal scales, robust methods (31) and the Diagonally Weighted Least Squares method (DWLS) were used (32). Fit indices included the Comparative Fit Index (CFI) (values ≥0.90 indicate acceptable fit; values ≥0.95 indicate good model fit) and the Root Mean Square Error of Approximation (RMSEA) (values ≤ 0.08 indicate acceptable model fit; values ≤ 0.05 indicate good model fit) [e.g., (33)]. To evaluate the fit difference between nested models, the differences between both the CFI and RMSEA indices (ΔCFI and ΔRMSEA, respectively) were used (values ≤ 0.01 in both the ΔCFI and an increasing <0.015 in the ΔRMSEA indicate non-significant differences between the models) [e.g., (34, 35). Fourth, the convergent validity of the MEMS subscales was reported with the Average Variance Extracted AVE, which should be > 0.50) (33), and the discriminant validity was obtained by squaring the correlation between the factors of the scale. Fifth, the correlations with the PIL-10 (meaning in life) and PANAS-P (positive affect) were analyzed to report the concurrent validity of the MEMS subscales. The correlations between the MEMS subscales and the BIS and PANAS-N (negative affect) were calculated to report the divergent validity. Sixth, the differences between men and women and between the age groups on the MEMS subscales were analyzed using the Mann-Whitney test and the Kruskal-Wallis test, respectively.

With the participants in the clinical sample, we performed four linear regression analyses with the participants diagnosed with mental disorders, taking the model composed of comprehension, purpose, and mattering as predictor variables and the BSI-18 depression subscale, BSI-18 anxiety subscale, general distress (BSI-18 overall score), and PANAS-P as dependent variables. For all these statistical analyses, the JASP 0.14.1 statistical software (36) was used. Interpretations of effect sizes were based on Cohen (37).

## Results

### Descriptive Statistics of the Scales Used in This Study

Table 1 shows the means, standard deviations, skewness, and kurtosis of the items on the MEMS, the McDonald's omega, and the item-rest correlations.

**Table 1:** Descriptive statistics and internal consistency of the scales used in this study in the non-clinical sample.

	**MEMS subscales**					
	**Comprehension**	**Purpose**	**Mattering**	**PIL-10**	**BSI**	**PANAS-P**	**PANAS-N**
*M*	28.39	30.26	26.41	53.94	23.45	36.90	26.84
*SD*	5.60	5.09	7.12	10.60	17.57	7.13	8.42
*Sk*	−0.94	−1.35	−0.78	−0.78	0.41	−0.42	−0.04
*K*	0.83	2.01	−0.09	0.46	−1.15	0.07	−0.73
$\bar{\omega }$	0.91	0.92	0.86	0.94	0.96	0.92	0.88

*N = 1,106. Sk = Skewness; K = Kurtosis. Skewness Standard Error was 0.07. Kurtosis Standard Error was 0.15*.

Table 2 shows the descriptive statistics, skewness, and kurtosis of the MEMS items, as well as the corrected item-total correlations, all of which showed values >0.40, indicating very good discrimination (38).

**Table 2:** Descriptive statistics of the MEMS items.

**MEMS subscale**	**Item**	**Statement**	***M***	***SD***	***Skw***	***K***	***Corrected r*_item-total_**
Comprehension	1	My life makes sense / Mi vida tiene sentido	6.05	1.193	−1.40	1.93	0.691
	7	I know what my life is about / Yo sé de qué trata mi vida	5.65	1.40	−1.11	1.00	0.804
	8	I can make sense of the things that happen in my life / Puedo construir un sentido de las cosas que pasan en mi vida	5.83	1.21	−1.09	1.11	0.798
	10	I understand my life / Comprendo mi vida	5.59	1.34	−0.95	0.60	0.840
	14	Looking at my life as a whole, things seem clear to me / Mirando mi vida como un todo, las cosas parecen evidentes	5.27	1.43	−0.65	−0.04	0.689
Purpose	3	I have aims in my life that are worth striving for / Tengo objetivos en mi vida por los que vale la pena luchar	6.30	1.05	−1.74	3.25	0.782
	5	I have certain life goals that guide me to keep going / Tengo ciertas metas en la vida que me obligan a seguir adelante	6.10	1.15	−1.50	2.44	0.792
	6	I have overarching goals that guide me in my life / Tengo objetivos globales que me guían en mi vida	5.99	1.21	−1.41	2.18	0.809
	9	I have goals goals in my life that are very important to me / Tengo metas y objetivos en mi vida muy importantes para mí	6.09	1.14	−1.40	2.02	0.855
	12	My direction in life in motivating to me / Mi sentido en la vida es motivador para mí	5.79	1.28	−1.20	1.37	0.744
Mattering	2	There is nothing special about my existence / No hay nada que haga especial mi existencia	5.93	1.72	−1.59	1.40	0.454
	4	Even a thousand year from now, it would still matter whether I existed of not / Incluso dentro de mil años, todavía importaría si yo existiera o no	4.71	2.06	−0.53	−0.95	0.701
	11	Whether my life ever existed matters even in the grand scheme of the universe / Si mi vida alguna vez existió, fue importante en el esquema general del universo	4.73	1.94	−0.55	−0.79	0.742
	13	I am certain that my life is of importance / Estoy seguro de que mi vida es importante	5.75	1.40	−1.25	1.28	0.714
	15	Even considering how big the universe is, I can say that my life matters / Incluso considerando lo grande que es el universo, puedo decir que mi vida importa	5.39	1.70	−1.09	0.45	0.762

*N = 1,106. Sk = Skewness; K = Kurtosis. In parentheses, the standard error. Skewness Standard Error was 0.07; Kurtosis Standard Error was 0.15*.

The average inter-item correlations of the MEMS subscales were higher than 0.30 and lower than 0.80: 0.658 for the Comprehension subscale, 0.702 for the Purpose subscale, and 0.545 for the Mattering subscale. That is, the MEMS subscales did not show homogeneity and multicollinearity problems (39).

### Structural Validity of the MEMS and Invariance Across Gender and Age Groups

**Figure 1:**
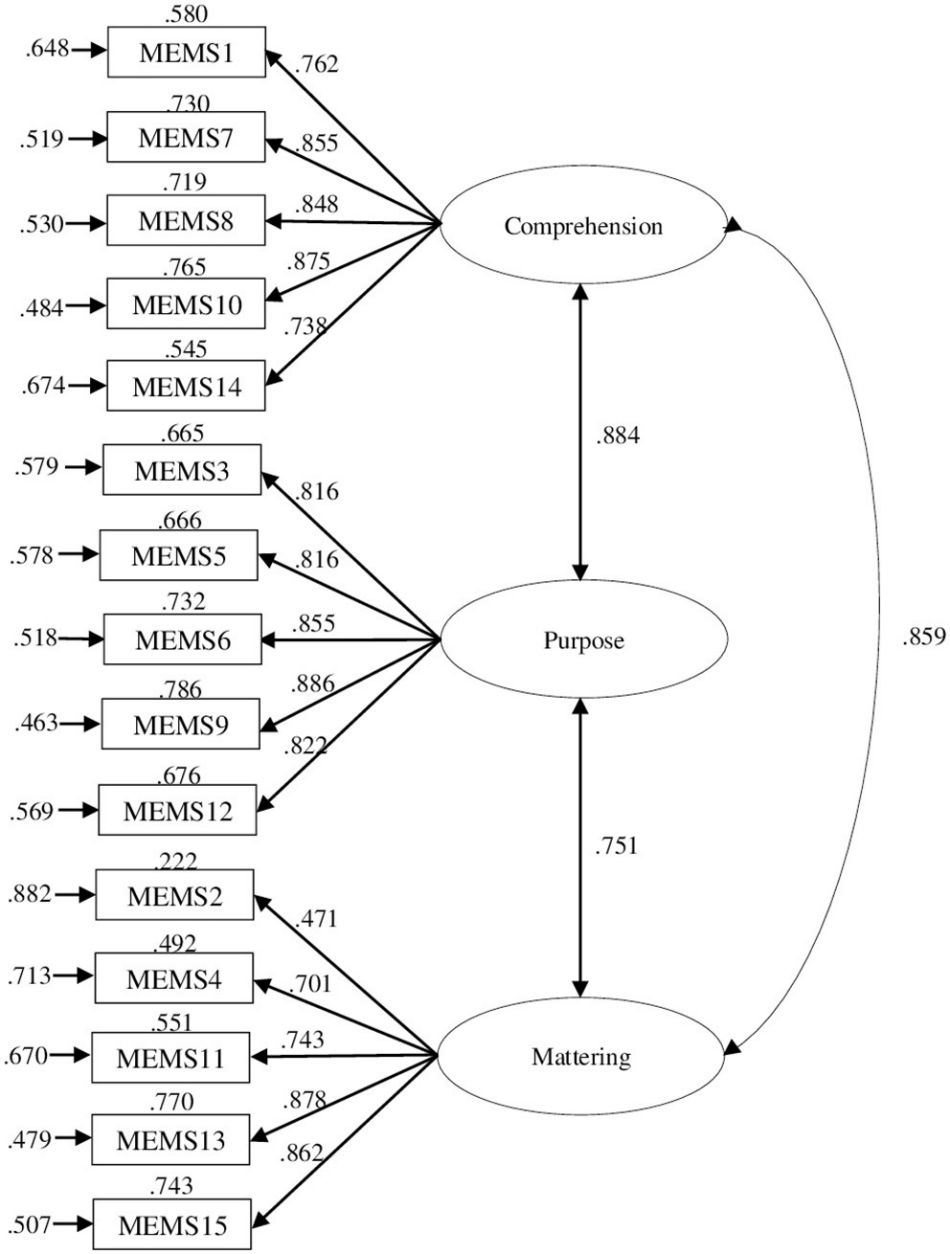
Model for the MEMS obtained in this study. Values at the top of each rectangle are R^2^ and values at the left of each rectangle are errors.

The MEMS showed a good fit: SB$\chi_{\left(87\right)}^{2}$ = 262.953, *p* < 0.001, CFI = 0.991, RMSEA = 0.043 [0.037, 0.049] (Figure 1). All parameters were significant, *p* < 0.05.

Configural invariance, metric invariance, scalar invariance, and strict invariance across gender groups, as well as configural invariance across age groups, were obtained (Table 3).

**Table 3:** Invariance model for the MEMS across gender and age groups.

	**Model**	**SBχ^2^ (*df*)**	***p***	**CFI**	**RMSEA [90% CI]**	**ΔSB**χ**^**2**^ (*df*)**	**ΔCFI**	**ΔRMSEA**
Gender	Baseline men	113.960 (87)	0.028	0.992	0.035 [0.012, 0.052]			
	Baseline women	175.093 (87)	0.000	0.995	0.034 [0.027, 0.042]			
	Configural	289.053 (174)	0.000	0.994	0.035 [0.027, 0.042]			
	Metric	304.980 (186)	0.000	0.994	0.034 [0.027, 0.041]	15.927 (12)	0.000	0.001
	Scalar	315.038 (198)	0.000	0.994	0.033 [0.026, 0.039]	10.058 (12)	0.000	0.001
	Strict	324.494 (213)	0.000	0.994	0.031 [0.024, 0.037]	9.456 (15)	0.000	0.002
Age	Baseline 18–24	63.340 (87)	0.974	1.000	0.000 [0.000, 0.000]			
	Baseline 25–41	124.919 (87)	0.005	0.996	0.029 [0.017, 0.040]			
	Baseline 42–83	70.903 (87)	0.895	1.000	0.000 [0.000, 0.015]			
	Configural	259.162 (261)	0.521	1.000	0.000 [0.000, 0.020]			
	Metric	395.885 (285)	0.000	0.994	0.033 [0.024, 0.040]	136.723 (24)	0.006	0.033
	Scalar	495.337 (309)	0.000	0.990	0.040 [0.034, 0.047]	99.452 (24)	0.004	0.007
	Strict	557.750 (339)	0.000	0.988	0.042 [0.036, 0.048]	62.413 (30)	0.002	0.002

### Construct Validity of the MEMS

Convergent validity. The AVE values were good, >0.50, for the MEMS subscales: Comprehension, AVE = 0.67; Purpose, AVE = 0.70; and Mattering-R, AVE = 0.64; indicating good convergent validity of these scales (33).

Discriminant validity. The squared correlations between the MEMS subscales were lower than the AVE values, indicating discriminant validity (40): Comprehension-Purpose, $r_{\text{s}}^{2}$ = 0.65; Comprehension-Mattering, $r_{\text{s}}^{2}$ = 0.59; and Purpose-Mattering, $r_{\text{s}}^{2}$ = 0.43.

### Concurrent Validity of the MEMS Subscales

The MEMS subscales showed strong, positive correlations with both the PIL-10 and PANAS-P, and small to intermediate, negative correlations with both the BSI and PANAS-N (37) (Table 4).

**Table 4:** Correlations between the MEMS subscales and the PIL-10, BIS, and PANAS scales.

	**MEMS subcale**		
	**Comprehension**	**Purpose**	**Mattering**
PIL-10	0.745^**^	0.726^**^	0.647^**^
BSI	−0.272^**^	−0.343^**^	−0.073^*^
PANAS-P	0.618^**^	0.599^**^	0.500^**^
PANAS-N	−0.229^**^	−0.282^**^	−0.070^*^

*Spearman's Rho was used*.

* *p < 0.05 (bilateral)*;

** *p < 0.01 (bilateral)*.

### Differences on the MEMS Between Gender and Age Groups

Because the Shapiro-Wilk test suggested a deviation from normality, the Mann-Whitney test for independent samples was used for the gender-related differences in the MEMS subscales. Women showed higher means than men on the three MEMS subscales. The difference was significant for the Purpose subscale (Table 5).

**Table 5:** Descriptive statistics for men and women in the MEMS subscales and Mann-Whitney test.

			**Descriptive statistics**			**Mann-Whitney test**		
**MEMS subscale**	**Group**	***N***	***M***	***SD***	***SE***	***W***	***p***	***r_***b***_***
Comprehension	Men	251	28.016	5.592	0.353	100846.000	0.152	−0.059
	Women	854	28.500	5.603	0.192			
Purpose	Men	251	29.618	5.027	0.317	93865.500	0.002	−0.124
	Women	854	30.451	5.096	0.174			
Mattering	Men	251	26.000	7.244	0.457	102443.500	0.285	−0.044
	Women	854	26.542	7.088	0.243			

*For the Mann-Whitney test, effect size is given by the rank biserial correlation, r_b_*.

For the differences in the MEMS subscales between age groups, because the normality test was significant, the non-parametric Kruskal-Wallis test was used for comparisons. Likewise, because Levene's test for equality of variances was significant for the MEMS subscales (Comprehension: *F*_(2, 1103)_ = 8.360, *p* < 0.01; Purpose: *F*_(2, 1103)_ = 3.404, *p* < 0.05; Mattering: *F*_(2, 1103)_ = 7.964, *p* < 0.01), the Games-Howell *post-hoc* test was used (Table 6).

**Table 6:** Descriptive statistics, Kruskal-Wallis test, and *post-hoc* test for the differences between groups in the MEMS subscales.

			**Descriptive statistics**		**Kruskal-Wallis test**			**Games-Howell** ***post-hoc*** **comparisons age groups**					
**MEMS subscale**	**Age group**	***N***	***M***	***SD***	***H***	***df***	***p***	**Comparison**	**Mean Difference**	***SE***	***t***	***df***	***p*_Tukey_**
Comprehension	15–24	298	26.46	5.74	59.527	2	0.000	15–24/25–41 years	−2.275	0.417	−5.461	615.525	0.000
	25–41	513	28.73	5.68				15–24/42+ years	−3.283	0.433	−7.590	572.991	0.000
	42+	295	29.74	4.75				25–41/42+ years	−1.008	0.373	−2.700	703.386	0.019
Purpose	15–24	298	29.73	5.04	17.207	2	0.000	15–24/25–41 years	−0.276	0.376	−0.735	652.018	0.743
	25–41	513	30.01	5.36				15–24/42+ years	−1.516	0.393	−3.861	585.134	0.000
	42+	295	31.25	4.51				25–41/42+ years	−1.240	0.353	−3.508	699.909	0.001
Mattering	15–24	298	23.12	7.47	89.959	2	0.000	15–24/25-41 years	−4.773	0.524	−9.112	566.433	0.000
	25–41	513	27.89	6.69				15–24/42+ years	−4.055	0.572	−7.095	579.420	0.000
	42+	295	27.17	6.41				25–41/42+ years	0.718	0.476	1.508	633.980	0.288

On both the Comprehension and Purpose subscales, the higher the age range, the higher the mean. On the Mattering subscale, the age group that showed the highest mean ranged from 25 to 41 years old, followed by the 42-year-old and up group and the 15–24-year-old group. Mean differences were significant for all the comparisons, except for those between the 15–24-year-old group and the 25–41-year-old group on the Purpose subscale, *p*_Tukey_ = 0.743, and between the 25–42-year-old group and the 42-year-old and up group on the Mattering subscale, *p*_Tukey_ = 0.288.

### The Predictive Role of Each MiL Dimension in Psychopathology in Participants With Mental Disorders

Regarding psychopathology of participants with mental disorders comprehension was highly and negatively associated with depression, and psychopathological distress. Purpose and mattering were highly and negatively associated with depression. Mattering was highly and negatively associated with depression, and psychopathological distress. Positive affect, was moderately and positively associated the three dimensions of MiL. The rest of the correlations can be seen in Table 7.

**Table 7:** Correlations between the variables studied in clinical participants.

	***M* (*SD*)**	**2**	**3**	**4**	**5**	**6**	**7**
1 Comprehension (MEMS)	24.93 (7.06)	0.78*	0.78*	−0.67^*^	−0.42^*^	−0.55^*^	0.45^*^
2 Purpose (MEMS)	28.62 (6.32)		0.76^*^	−0.64^*^	−0.36^*^	−0.48^*^	0.46^*^
3 Mattering (MEMS)	21.65 (8.12)			−0.67^*^	−0.46^*^	−0.56^*^	0.36^*^
4 Depression (BSI)	10.62 (6.42)				0.71^*^	0.86^*^	−0.19^*^
5 Anxiety (BSI)	9.86 (6.32)					0.93^*^	−0.12
6 General Psychopathology (BSI)	29.02 (16.79)						−0.01
7 Positive Affect (PANAS)	31.92 (5.30)						

*MEMS, Multidimensional Existencial Meaning Scale; BSI, Brief Simptoms Scale; PANAS, Positive Negative Affect Schedule*.

* *p < 0.01*.

Depressive symptoms. The model composed of comprehension, purpose, and mattering was a significant predictor of depressive symptoms (*r*^2^ = 0.52, *F*_(3, 84)_ = 30.32, *p* < 0.001). The proposed model accounted for 52 % of the variance in depressive symptoms. When the individual contribution of each dimension of MiL was analyzed, mattering was the most strongly associated with depressive symptoms, followed by comprehension (Table 8).

**Table 8:** Regression analyses predicting depression, anxiety, general psychopathology, and positive affect in participants with mental disorders.

**Dependent variable**	**Predictor variable**	**B standardized**	***SE***	***t***
Depressive symptoms	Comprehension	−0.248	0.124	−2.001*
	Purpose	−0.187	0.134	−1.396
	Mattering	−0.256	0.104	−2.467*
Anxiety	Comprehension	−0.164	0.155	−1.056
	Purpose	0.065	0.168	0.385
	Mattering	−0.287	0.130	−2.204*
General psychopathology	comprehension	−0.676	0.377	−1.792
	Purpose	−0.003	0.408	−0.007
	Mattering	−0.700	0.316	−2.214*
Positive affect	Comprehension	0.213	0.129	1.652
	Purpose	0.269	0.139	1.931*
	Mattering	0.068	0.108	−0.626

*p < 0.01; **p < 0.001.

Anxiety symptoms. The proposed model was a significant predictor of anxiety symptoms (*r*^2^ = 0.22, *F*_(3, 84)_= 8.052, *p* < 0.001). The proposed model accounted for 22 % of the variance in anxiety symptoms. When the individual contribution of each dimension of MiL was analyzed, only mattering was a significant predictor of anxiety symptoms (Table 8).

Psychopathological distress. The proposed model was a significant predictor of general psychopathology (BSI-18 total scale) (*r*^2^ = 0.35, *F*_(3, 84)_ = 14.88, *p* < 0.001). The proposed model accounted for 35% of the variance in general psychopathology. When the individual contribution of each dimension of MiL was analyzed, only mattering was a significant predictor of anxiety symptoms (Table 8).

Positive affect. Finally, the proposed model was a significant predictor of positive affect (*r*^2^ = 0.24, *F*_(3, 84)_ = 8.807, *p* < 0.001). The proposed model accounted for 24% of the variance in positive affect. When the individual contribution of each dimension of MiL was analyzed, only purpose was a significant predictor of positive affect (Table 8).

## Discussion

The results obtained in the present study indicate that the three-factor model for the MEMS (comprehension, purpose, and mattering) showed an acceptable fit, similar to the original structure (22), in a sample of Spanish participants. The scale showed good internal consistency in the three factors, with acceptable indexes. The MEMS showed full invariance across gender groups, whereas only configural invariance across age groups was obtained. Moreover, our results showed good convergent validity and discriminant validity of the three MEMS subscales. Regarding concurrent validity, the MEMS subscales showed high, positive correlations with both the PIL-10 and PANAS-P, and low to medium, negative correlations with both the BSI-18 and PANAS-N.

Thus, our results support the original three-factor structure (22), and they coincide with Gerymski and Krok's study (23), which analyzed the factorial structure of the MEMS questionnaire in Poland. However, in the Polish validation, although the factorial structure with three factors was confirmed, the scale was reduced to nine items. In our study, in addition to confirming the original factorial structure, the same items were maintained in each factor as in the original. Thus, this is the first study to fully confirm the MEMS factorial structure in a sample of non-American participants.

Women showed higher means than men on the three MEMS subscales. The difference was significant for the purpose subscale. On the one hand, these results are consistent with those found in Spanish validation studies of one-dimensional MiL measures, such as the PIL, where women had higher scores than men (41). On the other hand, in studies of one-dimensional MiL measurements carried out in Anglo-Saxon samples, no differences were found in the MiL constructs depending on gender (1, 2). Thus, the differences in MiL scores between men and women could be due to sociocultural factors. Future studies should investigate the role of social, cultural, and religious factors in the elaboration of MiL, and analyze the properties of MEM in non-Western samples, such as African or Asian populations) (42).

On both the comprehension and purpose subscales, the higher age ranges showed higher scores. On the mattering subscale, the age group that showed the highest mean was the 25–41-year-old group, followed by the 42-and-up group and then the 15–24-year-old group. These results are similar to other studies that found that MiL assessed with the MLQ (presence of meaning) was positively associated with age (2), confirming that MiL and its dimensions is positively associated with age.

After confirming the factorial structure of the MEMS in the Spanish population, the second objective of the present study was to analyze which dimension of the MiL was most associated with different types of psychopathology, distress, and positive affect in a sample of participants diagnosed with mental disorders who were undergoing psychological or pharmacological treatment. Our results indicate that the different MiL dimensions are differentially associated with different symptoms of psychopathology and positive affect. Depressive symptoms were more robustly associated with both the mattering and comprehension dimensions. Symptoms of anxiety were associated with the mattering dimension. Psychopathological distress (composed of symptoms of anxiety, depression, and somatization together) was predicted primarily by the mattering dimension. Thus, in our clinical sample, the mattering dimension had a higher association with psychopathology than comprehension or purpose. These results support previous studies that found that mattering was negatively associated with suicide ideation (43) and other studies that showed the positive influence of mattering on mental health (44, 45). However, these results differ from other studies in non-clinical populations where the comprehension dimension showed a stronger association with psychopathology than the purpose and mattering dimensions [e.g., (22)]. Mattering, consists of two dimensions: interpersonal mattering and societal mattering (46). Interpersonal mattering refers to a person's perception that he or she matters to others and societal mattering is “the feeling of making a difference in the broader scheme of sociopolitical events—of feeling that one's thoughts and actions have an impact, create ripples, are felt” (46). In people with mental disorders, both dimensions of mattering could be impaired. Previous studies about mental health symptoms and socio-economic conditions found that people with mental disorders showed poor social support, high unemployment rate (61.4% in our sample), and have functional disability (47).

Finally, positive affect was predicted by the purpose dimension. The association between purpose and positive affect obtained in our clinical sample coincides with the results of previous studies with non-clinical populations (22).

Our results support previous studies that demonstrated that multidimensional questionnaires to evaluate MiL showed better psychometric properties and better results when related to well-being or psychopathology variables than one-dimensional models (2).

These results have important clinical implications. Although previous studies found a strong association between MiL and psychopathology (9, 10, 14, 15), these studies considered MiL as a one-dimensional construct, which made it difficult to know what the most important MiL components were and, thus, develop specific interventions focused on the most important MiL dimensions for each patient. On the one hand, our results suggest that when distress symptoms, especially anxiety and depressive symptoms, are present, it would be helpful to carry out psychological interventions that focus on the mattering dimension. On the other hand, to increase positive affect, our results suggest that it might be necessary to intervene in the purpose dimension. Review studies suggest that meaning-centered therapies strongly improve quality of life and reduce psychological distress, particularly in transitional moments in life, participants with chronic illnesses, and patients with low meaning in life (48).

Our study has several limitations. First, the sampling method employed snowball techniques to recruit participants through main social networks (Facebook, WhatsApp, Twitter, Linkedin, and Instagram). Therefore, the sample may not be representative of the general Spanish population. Furthermore, although the Spanish version of the MEMS has shown adequate psychometric characteristics, an analysis of test-retest reliability could not be performed. Future studies should be longitudinal and confirm the test-retest reliability of the MEMS. To recruit the clinical sample, no diagnosis was made by psychologists specialized in clinical psychology or psychiatrists, and so the diagnoses were not confirmed. The results must be understood in terms of association, rather than prediction or causality, because the study was cross-sectional and not experimental.

Despite the aforementioned limitations, our study suggests that the MEMS is an adequate instrument to assess the three dimensions of MiL (comprehension, purpose, and mattering) in Spanish-speaking participants. Moreover, our results suggest that it is necessary to assess MiL from a multidimensional perspective. The present study tries to make a modest contribution to this line of research.

## Data Availability Statement

The raw data supporting the conclusions of this article will be made available by the authors, without undue reservation.

## Ethics Statement

The studies involving human participants were reviewed and approved by Valencian University Ethics Committee. The patients/participants provided their written informed consent to participate in this study.

## Author Contributions

JM drafted the manuscript with important contributions from JG-A and VG. JM in collaboration with JG-A, VG, RB, SR and MT-I, designed the study and participated in each of its phases. JG-A, VG, RB, SR and MT-I translated and adapted the MEMS. All authors participated in the review and revision of the manuscript and have approved the final manuscript to be published.

## Funding

This research received any specific grant from Consejeria de Innovación, Universidades, Ciencia y Sociedad Digital: Subvenciones para Grupos de Investigación Consolidables – AICO/2021 Ref: 20210862.

## Conflict of Interest

The authors declare that the research was conducted in the absence of any commercial or financial relationships that could be construed as a potential conflict of interest.

## Publisher's Note

All claims expressed in this article are solely those of the authors and do not necessarily represent those of their affiliated organizations, or those of the publisher, the editors and the reviewers. Any product that may be evaluated in this article, or claim that may be made by its manufacturer, is not guaranteed or endorsed by the publisher.
